# A Dipeptidyl Peptidase-4 Inhibitor Suppresses Macrophage Foam Cell Formation in Diabetic *db/db* Mice and Type 2 Diabetes Patients

**DOI:** 10.1155/2018/8458304

**Published:** 2018-12-09

**Authors:** Michishige Terasaki, Munenori Hiromura, Yusaku Mori, Kyoko Kohashi, Hideki Kushima, Masakazu Koshibu, Tomomi Saito, Hironori Yashima, Takuya Watanabe, Tsutomu Hirano

**Affiliations:** ^1^Department of Medicine, Division of Diabetes, Metabolism, and Endocrinology, Showa University School of Medicine, Tokyo, Japan; ^2^Laboratory of Cardiovascular Medicine, Tokyo University of Pharmacy and Life Sciences, Hachioji City, Tokyo, Japan

## Abstract

Dipeptidyl peptidase-4 (DPP-4) inhibitors could have antiatherosclerotic action, in addition to antihyperglycemic roles. Because macrophage foam cells are key components of atherosclerosis, we investigated the effect of the DPP-4 inhibitor teneligliptin on foam cell formation and its related gene expression levels in macrophages extracted from diabetic *db/db* (*C57BLKS/J Iar -+Lepr^db^/+Lepr^db^*) mice and type 2 diabetes (T2D) patients ex vivo. We incubated mouse peritoneal macrophages and human monocyte-derived macrophages differentiated by 7-day culture with oxidized low-density lipoprotein in the presence/absence of teneligliptin (10 nmol/L) for 18 hours. We observed remarkable suppression of foam cell formation by teneligliptin treatment *ex vivo* in macrophages isolated from diabetic *db/db* mice (32%) and T2D patients (38%); this effect was accompanied by a reduction of CD36 (*db/db* mice, 43%; T2D patients, 46%) and acyl-coenzyme A: cholesterol acyltransferase-1 (ACAT-1) gene expression levels (*db/db* mice, 47%; T2D patients, 45%). Molecular mechanisms underlying this effect are associated with downregulation of CD36 and ACAT-1 by teneligliptin. The suppressive effect of a DPP-4 inhibitor on foam cell formation in T2D is conserved across species and is worth studying to elucidate its potential as an intervention for antiatherogenesis in T2D patients.

## 1. Introduction

Type 2 diabetes (T2D) is well known to accelerate the clinical course of atherosclerosis, a condition associated with arterial endothelial dysfunction and several metabolic abnormalities. During the early stage of atherosclerosis, subendothelial accumulation of lipid-laden macrophage-derived foam cells occurs. Accumulation of cholesterol esters in macrophages is a hallmark of foam cell formation, which depends on the uptake of oxidized low-density lipoprotein (ox-LDL) via CD36 [1]. It is decreased by the efflux of free cholesterol mediated by ATP-binding cassette transporter (ABC) A1 and ATP-binding cassette subfamily G member 1 (ABCG1) [1]. To protect cells from toxicity resulting from excessive free cholesterol accumulation, the free cholesterol is esterified to cholesteryl ester by acyl-coenzyme A: cholesterol acyltransferase-1 (ACAT-1) [2]. ACAT-1 thereby contributes to foam cell formation by promoting cholesteryl ester accumulation in macrophages.

Dipeptidyl peptidase-4 (DPP-4) inhibitors have been widely used to lower glucose levels for the treatment of T2D patients. DPP-4 is an enzyme that degrades active glucagon-like peptide-1 (GLP-1) and active glucose-dependent insulinotropic peptide (GIP), incretins that are secreted on food intake. DPP-4 inhibitors act in concert to stimulate insulin secretion, leading to improved glucose levels. Reportedly, in addition to their antihyperglycemic roles, DPP-4 inhibitors have antiatherosclerotic actions in various animal models [3–8]. We previously demonstrated in *in vivo* studies that GLP-1, GIP, and a DPP-4 inhibitor, respectively, prevented the acceleration of atherosclerosis via suppression of foam cell formation regulated by CD36 and ACAT-1 in macrophages isolated from mice [3, 9]. Although DPP-4 inhibitors can prevent the accumulation of monocyte/macrophage foam cells in the atherosclerotic plaque formation in animals, their precise effects on macrophage foam cell formation in diabetic rodents and T2D patients remain unclear, especially in ex vivo studies. Therefore, the present study is aimed at elucidating the potential effect of a DPP-4 inhibitor on cardiovascular risk markers, foam cell formation in macrophages, and associated gene expression levels in macrophages isolated from diabetic *db/db* mice and T2D patients *ex vivo*.

## 2. Materials and Methods

### 2.1. Animal Experiments

This study was conducted in strict accordance with the recommendations in the *Guide for the Care and Use of Laboratory Animals* of the National Institutes of Health. The protocol was approved by the Institutional Animal Care and Use Committee of Showa University (permit number: 07005). All surgeries and sacrifices were performed under general anesthesia using isoflurane and with efforts to minimize suffering. Seven 7-week-old male *db/db* (*C57BLKS/J Iar -+Lepr^db^/+Lepr^db^*) mice, a mouse model of T2D, and seven 7-week-old male *db/db misty* (*C57BLKS/J Iar -m+/m+*) mice were purchased from Sankyo Labo Service (Tokyo, Japan) and kept on standard rodent chow. At 13 weeks of age, peritoneal macrophages and blood samples were collected, as previously described [3, 4, 9]. In brief, after intraperitoneal injection of thioglycolate broth, the peritoneal cells were isolated. The cells were seeded onto 3.5 cm dishes (3 × 10^6^ cells/dish) and allowed to adhere to the dish, followed by incubation for 1 hour at 37°C in 5% CO_2_ in RPMI 1640 medium containing 5% fetal bovine serum (FBS), 100 U/mL streptomycin, and 100 U/mL penicillin. After the completion of the incubation, the adherent cells that were identified as peritoneal macrophages were used for a cholesterol esterification assay, reverse transcription polymerase chain reaction (RT-PCR), or immunohistochemistry.

### 2.2. Measurement of Mouse Plasma

The blood samples collected after a 12-hour fast were used for analysis. Fasting blood glucose (FBG) levels were measured using a Stat strip XP2 dextrometer (Nipro, Osaka, Japan). Hemoglobin A1c (HbA1c) levels were measured using an A1CNow Plus kit (Bayer, Frankfurt, Germany) before sacrificing the animals. Plasma levels of total cholesterol (Total-C), high-density lipoprotein cholesterol (HDL-C), and triglyceride were measured by enzymatic methods (Wako, Osaka, Japan). Plasma levels of insulin were determined by enzyme-linked immunosorbent assay (ELISA) using an ultrasensitive mouse insulin ELISA kit (Morinaga, Kanagawa, Japan). In addition, systolic and diastolic blood pressures (SBP and DBP, respectively) were measured on the day of sacrifice in the fasting state by the tail-cuff method (Model MK-2000ST, Muromachi Kikai, Tokyo, Japan) [3, 4, 9]. In a subset of animals, oral glucose tolerance tests (OGTTs) were conducted, as previously described [4, 10].

### 2.3. Immunofluorescent Staining of Peritoneal Macrophages in Mice

For immunohistochemistry, an anti-F4/80 antibody (Alexa Fluor® 647, Ab204467; Abcam, Cambridge, England) at a concentration of 1 : 250 was added to RPMI 1640 medium with 5% FBS, which contained 10 *μ*g/mL Dil-ox-LDL (Highland Technology Center, MD, USA) with/without 10 nmol/L teneligliptin [11], and the incubation was continued for 18 hours at 37°C in 5% CO_2_. After washing, the stained macrophages were mounted in VECTASHIELD HardSet Mounting Medium with DAPI (H-1500, Vector Laboratories, CA, USA) and imaged with a BZ-X710 microscope/software (Keyence, Osaka, Japan).

### 2.4. Human Experiments

The study protocol was approved by the Ethics Committee of the Showa University School of Medicine (Tokyo, Japan; approval number: 956). Written informed consent was obtained from all T2D patients who had attended at Showa University Hospital and healthy volunteers after receiving a clear explanation of the study protocol. The study was designed in compliance with the Declaration of Helsinki.

The patients were diagnosed with T2D as their blood glucose control specified in the *Treatment Guide for Diabetes* (Japan Diabetes Society 2010) was inadequately controlled (HbA1c ≥ 7.0%) according to the National Glycohemoglobin Standardization Program (NGSP), despite insulin therapy in addition to dietary/exercise therapy or concomitant oral drug therapy other than DPP-4 inhibitors over ≥12 weeks. The healthy volunteers were free of diabetes mellitus and were taking no medications for diabetes.

After a 12-hour overnight fast, blood samples were collected from four 38–81-year-old male T2D patients and four 36–64-year-old healthy male volunteers. Human peripheral mononuclear cells were isolated from their blood. Monocytes purified using anti-CD14 antibody-conjugated magnetic microbeads (Miltenyi Biotec, CA, USA) were seeded onto 3.5 cm dishes (1 × 10^6^ cells/dish). The adherent monocytes were incubated at 37°C in 5% CO_2_ for 7 days in RPMI 1640 medium supplemented with 10% pooled human serum (HS), 100 U/mL streptomycin, and 100 U/mL penicillin to induce differentiation to macrophages, which were subsequently used for the cholesterol esterification assay or RT-PCR.

### 2.5. Measurement of Characteristics and Biochemical Parameters of the Human Subjects

We used the body mass index (BMI; the weight in kilograms divided by the square of the height in meters) as an index of obesity. SBP and DBP were measured twice with a mercurial sphygmomanometer (YAMASU, KENZMEDICO, Saitama, Japan). FBG, HbA1c (NGSP), LDL-C, HDL-C, and triglyceride were measured by conventional direct methods.

### 2.6. Cholesterol Esterification Assay in Macrophages Isolated from Mice and Humans

The cholesterol esterification assay was conducted as previously described [3, 4, 9]. In brief, mouse peritoneal macrophages or human macrophages differentiated by 7-day culture were incubated at 37°C in 5% CO_2_ for 18 hours in RPMI 1640 medium containing 10 *μ*g/mL ox-LDL with 0.1 mmol/L [^3^H] oleate in the presence/absence of 10 nmol/L teneligliptin. The cellular lipids were extracted, and the radioactivity of the cholesterol [^3^H] oleate was determined by thin-layer chromatography.

### 2.7. Gene Expression Levels in Macrophages Isolated from Mice and Humans

Adherent macrophages were incubated at 37°C in 5% CO_2_ for 18 hours with RPMI 1640 medium with/without 10 nmol/L teneligliptin ex vivo. Total RNA was isolated from mouse peritoneal macrophages or human macrophages differentiated by 7-day culture using a QIAGEN reagent (Hilden, Germany). Gene expression was assessed by real-time RT-PCR using the TaqMan Gene Expression Assay and Sequence Detection System (ABI PRISM 7900, Life Technologies, CA, USA), as previously described [9, 10].

### 2.8. Statistical Analysis

Values are expressed as mean ± SEM. Statistical analyses were performed by unpaired *t*-test when two groups were analyzed and ANOVA with Tukey's post hoc test when more than two groups were analyzed. Categorical variables were compared by the chi-square test. All analyses were performed by GraphPad Prism version 7.0 software (GraphPad Software, CA, USA). The significance level was defined as *p* < 0.05.

## 3. Results

### 3.1. Characteristics and Laboratory Data of Mice and Humans

Table 1 shows the characteristics and laboratory data of the diabetic *db/db* mice and *db/db misty* mice. Compared with the *db/db misty* mice, these diabetic *db/db* mice exhibited the classical features of the mouse T2D model, such as severe hyperglycemia (HbA1c, 8.3 ± 0.2% vs. 4.2 ± 0.1%; FBG, 432 ± 26 mg/dL vs. 107 ± 5 mg/dL), hyperphagia (food intake, 4.5 ± 0.2 g/day vs. 3.1 ± 0.1 g/day), high body weight gain (43.8 ± 0.6 g vs. 25.4 ± 0.3 g), high insulin concentration (2.48 ± 0.32 ng/dL vs. 0.27 ± 0.03 ng/dL), and high triglyceride levels (134 ± 16 mg/dL vs. 91 ± 3 mg/dL). Although Total-C and HDL-C levels were slightly higher in the *db/db* mice than in the *db/db misty* mice, the differences were not significant. Consistent with the high FBG and HbA1c levels, the diabetic *db/db* mice showed higher glucose loading after OGTT and area under the curve than the *db/db misty* mice (1215 ± 85 mg/dL × hour vs. 276 ± 3 mg/dL × hour).

Table 2 summarizes the characteristics and plasma biochemical parameters of the four T2D patients and four healthy volunteers. In spite of insulin therapy in addition to dietary/exercise therapy or concomitant oral drug therapy other than DPP-4 inhibitors over ≥12 weeks, the T2D patients had high glucose levels than the healthy volunteers (HbA1c, 8.3 ± 0.2% vs. 5.4 ± 0.2%; FBG, 189 ± 24 mg/dL vs. 93 ± 2 mg/dL), and the duration of T2D was 19 ± 6 years. The T2D patients had progressive diabetic complications such as retinopathy, nephropathy, coronary artery disease, and peripheral artery disease. The use of oral antidiabetic, lipid-lowering, and antihypertensive drugs and the dose of insulin were described. Supplementary Table 1 lists the parameters of the individual T2D patients and healthy volunteers.

### 3.2. Coexpression of F4/80 and Dil-ox-LDL in Mouse Peritoneal Macrophages

**Figure 1:**
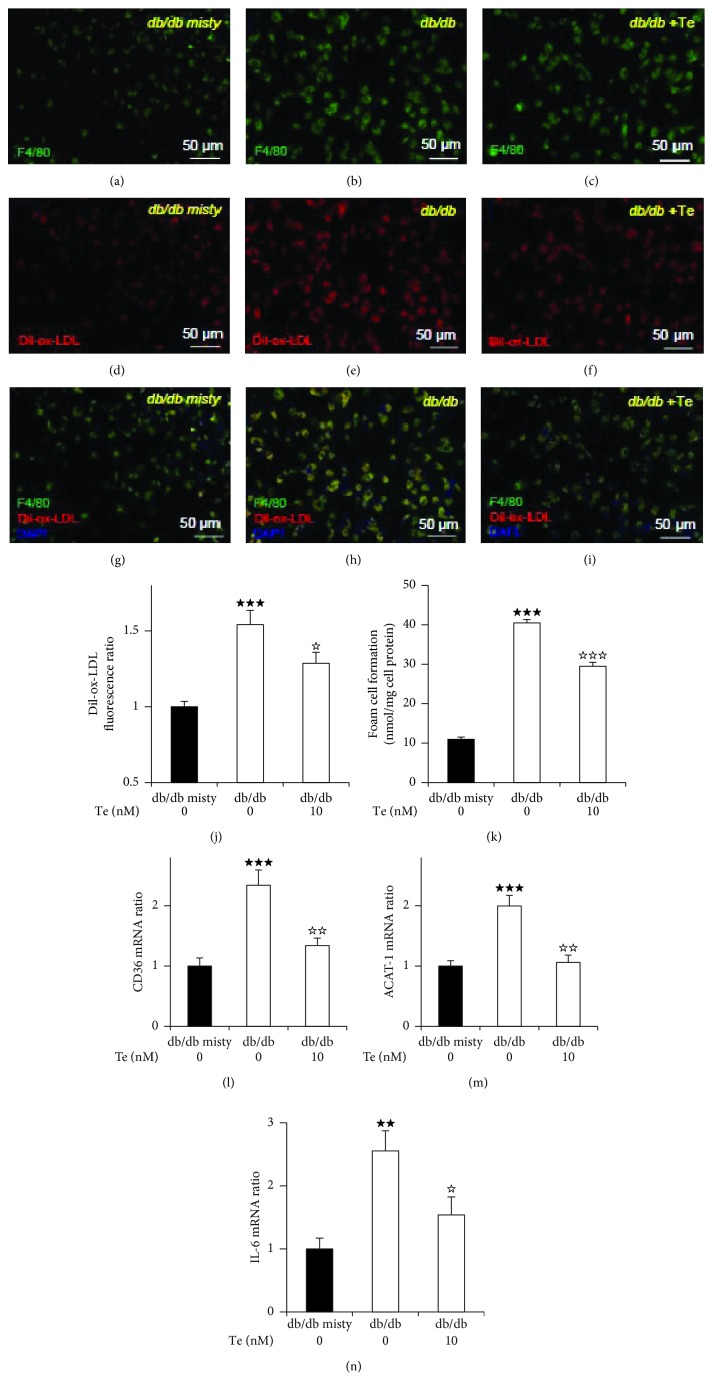
Effects of the dipeptidyl peptidase-4 (DPP-4) inhibitor teneligliptin on oxidized low-density lipoprotein accumulation and its related gene expression levels in macrophages extracted from diabetic *db/db* mice and *db/db misty* mice at 13 weeks old. Four days after an intraperitoneal injection of thioglycolate, the exudate peritoneal cells were isolated from diabetic *db/db* (*C57BLKS/J Iar -+Lepr^db^/+Lepr^db^*) mice and *db/db misty* (*C57BLKS/J Iar -m+/m+*) mice at 13 weeks of age. For cholesterol accumulation, adherent macrophages were incubated at 37°C in 5% CO_2_ for 18 hours with RPMI 1640 medium containing 5% fetal bovine serum (FBS) and 10 *μ*g/mL oxidized low-density lipoprotein (ox-LDL) in the presence of 0.1 mmol/L [^3^H] oleate or 10 *μ*g/mL Dil-ox-LDL, which were added with/without 10 nmol/L teneligliptin (Te) ex vivo (a-k). For gene expression levels, adherent macrophages were incubated at 37°C in 5% CO_2_ for 18 hours with RPMI 1640 medium with 5% FBS in the presence/absence 10 nmol/L teneligliptin ex vivo without the addition of ox-LDL (l-n). (a-i) Representative immunofluorescent staining images of the peritoneal macrophages isolated from diabetic *db/db* mice or *db/db misty* mice. F4/80-expressing cells were in green, Dil-ox-LDL-stained cells were in red, and DAPI-stained cells were in blue. Scale bars represent 50 *μ*m. (j) Relative fluorescence ratio per area of Dil-ox-LDL in peritoneal macrophages isolated from mice was imaged and analyzed with a BZ-X710 microscope/software. (k) Foam cell formation in exudate peritoneal macrophages isolated from diabetic *db/db* mice and *db/db misty* mice. The cellular lipids were extracted, and the radioactivity of the cholesterol [^3^H] oleate was determined by thin-layer chromatography. (l-n) Gene expression levels related to foam cell formation in peritoneal macrophages isolated from diabetic *db/db* mice and *db/db misty* mice. Gene expression levels of CD36 (l), acyl-coenzyme A: cholesterol acyltransferase-1 (ACAT-1) (m), and interleukin-6 (IL-6) (n) and the association with GAPDH were analyzed by real-time RT-PCR without the addition of ox-LDL. Data information: *n* = 7*db/db misty* mice, *n* = 7*db/db* mice, and *n* = 7*db/db* mice with ex vivo teneligliptin treatment. Results are presented as mean values ± SEM and analyzed with one-way ANOVA: ^★★★^ *p* < 0.005, ^★★^ *p* < 0.01 vs. *db/db misty* mice. ^☆☆☆^ *p* < 0.005, ^☆☆^ *p* < 0.01, ^☆^ *p* < 0.05 vs. *db/db* mice without teneligliptin.

We evaluated whether F4/80 and Dil-ox-LDL were expressed in mouse peritoneal macrophages. Immunofluorescent staining showed that F4/80 and Dil-ox-LDL were mainly coexpressed in the cytoplasm of the mouse peritoneal macrophages (Figures 1(a)–1(i)), suggesting that ox-LDL accumulates in mouse macrophages. The Dil-ox-LDL relative fluorescence ratio per area increased in the diabetic *db/db* mice by 1.54 times compared with that in the *db/db misty* mice, which was reduced by 18% by ex vivo treatment with teneligliptin in the diabetic *db/db* mice (Figure 1(j)).

### 3.3. Cholesterol Ester Accumulation in Macrophages Isolated from Mice and Humans

**Figure 2:**
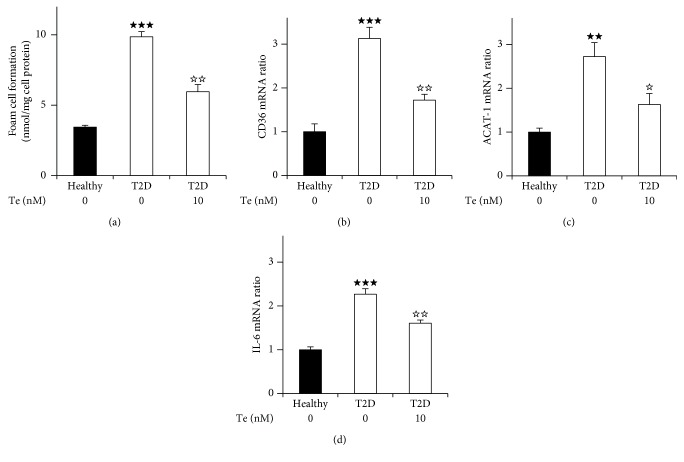
Effects of teneligliptin on foam cell formation and its related gene expression levels in monocyte-derived macrophages isolated from type 2 diabetes (T2D) patients and healthy volunteers. Human peripheral mononuclear cells were isolated from four type 2 diabetes (T2D) patients and four healthy volunteers. Monocytes purified using anti-CD14 antibody-conjugated magnetic microbeads were seeded onto dishes. Adherent monocytes were incubated at 37°C in 5% CO_2_ for 18 hours with RPMI 1640 medium containing 10% human serum (HS) for 7 days to induce differentiation to macrophages, which were in the presence/absence of 10 nmol/L teneligliptin. (a) Human macrophages differentiated by 7-day culture were incubated at 37°C in 5% CO_2_ for 18 hours in RPMI 1640 medium containing 10% HS with 10 *μ*g/mL ox-LDL and 0.1 mmol/L [^3^H] oleate in the presence/absence of 10 nmol/L teneligliptin *ex vivo*. The cellular lipids were extracted, and the radioactivity of the cholesterol [^3^H] oleate was determined by thin-layer chromatography. (b-d) Gene expression levels related to foam cell formation in monocyte-derived macrophages isolated from T2D patients and healthy volunteers. Adherent macrophages were incubated at 37°C in 5% CO_2_ for 18 hours with RPMI 1640 medium containing 10% HS in the presence/absence of 10 nmol/L teneligliptin *ex vivo.* Gene expression levels of CD36 (b), ACAT-1 (c), and IL-6 (d) and the association with GAPDH were analyzed by real-time RT-PCR without the addition of ox-LDL. Data information: *n* = 4 healthy volunteers, *n* = 4 T2D patients, and *n* = 4 T2D patients with *ex vivo* teneligliptin treatment after isolation. Results are presented as mean values ± SEM and analyzed with one-way ANOVA: ^★★★^ *p* < 0.005, ^★★^ *p* < 0.01 vs. health volunteers. ^☆☆^ *p* < 0.01, ^☆^ *p* < 0.05 vs. T2D patients without *ex vivo* teneligliptin treatment.

To determine whether teneligliptin regulated foam cell formation in macrophages ex vivo, we assessed the effect of teneligliptin on ox-LDL-induced foam cell formation in the peritoneal macrophages of the diabetic *db/db* mice and monocyte-derived macrophages differentiated by 7-day culture isolated from the T2D patients. The number of exudate peritoneal cells and morphological cell characteristics did not significantly differ among the groups. ox-LDL-induced cholesterol ester accumulation, which indicated the extent of foam cell formation, was approximately four times higher in the macrophages isolated from the diabetic *db/db* mice than in those from the *db/db* misty mice. This enhanced foam cell formation in the diabetic *db/db* mice was significantly suppressed by 32% by ex vivo treatment with teneligliptin (Figure 1(k)). On the other hand, in humans, the extent of macrophage foam cell formation in the T2D patients was approximately three times higher than that in healthy volunteers. Interestingly, we observed a remarkable decrease of 38% in the extent of macrophage foam cell formation by *ex vivo* treatment with teneligliptin in T2D patients (Figure 2(a)). In addition, we showed the extent of macrophage foam cell formation in individual healthy volunteers (Supplementary Figure 1, a-d), T2D patients (Supplementary Figure 1, e-h), and T2D patients with *ex vivo* teneligliptin treatment after isolation (Supplementary Figure 1, e'-h'). We extracted 4–5 dishes of adhered monocyte-derived macrophages in each group (Supplementary Figure 1, a-h'). The background characteristics and plasma biochemical parameters of individual T2D patients and healthy volunteers are also listed (Supplementary Table 1, a-h).

### 3.4. Changes in Gene Expression Associated with Macrophage Foam Cell Formation in Mice and Humans

To elucidate the molecular mechanisms underlying the reductive effect of teneligliptin on foam cell formation in macrophages isolated from the diabetic *db/db* mice and T2D patients, we examined gene expression levels associated with foam cell formation in macrophages. CD36 and ACAT-1 gene expression levels in macrophages isolated from the diabetic *db/db* mice and T2D patients were approximately 2–3 times higher than those from the *db/db misty* mice and healthy controls, respectively. *Ex vivo* teneligliptin treatment lowered the gene expression levels of CD36 (*db/db* mice, 43%; T2D patients, 46%) and ACAT-1 (*db/db* mice, 47%; T2D patients, 45%). In addition, the gene expression levels of the proinflammatory cytokine interleukin-6 (IL-6) in macrophages showed similar changes (*db/db* mice, 42%; T2D patients, 33%) (Figures 1(l)–1(n) and 2(b)–2(d)).

## 4. Discussion

To the best of our knowledge, the present ex vivo study is the first to demonstrate the suppressive effect of a DPP-4 inhibitor on foam cell formation in macrophages isolated from diabetic *db/db* mice and T2D patients. This study showed that the extent of macrophage foam cell formation in diabetic mice and T2D patients significantly increased compared with that in nondiabetic mice and healthy volunteers, which was accompanied by enhanced CD36 and ACAT-1 expression levels. There have been several supportive studies showing that CD36 and ACAT-1 expression or protein was highly expressed in monocytes/macrophages from diabetic mice and T2D patients [12–14]. The exact mechanism of hyperglycemia-induced enhancement of CD36 and ACAT-1 remains unknown; however, hyperglycemia-mediated accumulation of reactive oxygen species has the possibility to induce proinflammatory signaling, including p38 mitogen-activated protein kinase, leading to the induction of the nuclear transcription factor-kappaB (NF-*κ*B) signaling pathway and subsequent increase in CD36 expression [15]. Another key factor, ACAT-1, a rate-limiting enzyme for the esterification of free cholesterol from ox-LDL, plays a crucial role in foam cell formation in macrophages characterized by cholesterol ester accumulation [16]. Because ACAT-1 expression is regulated by free cholesterol availability inside cells [2], the changes of ACAT-1 expression levels might be a consequence of changes in CD36 levels.

We previously showed in *in vivo* studies that CD36 and ACAT-1 levels in mouse macrophages were decreased by the treatment with GLP-1, GIP, or DPP-4 inhibitors [3, 9]. In addition, because diabetic *db/db* mice do not accelerate visible atherosclerotic lesions, macrophage foam cell formation was used as a surrogate marker for atherosclerosis in our previous studies [4, 10]. Dai et al. reported that DPP-4 inhibitors directly repress foam cell formation in THP-1 macrophages by inhibiting the scavenger receptor CD36 *in vitro*[17], indicating that DPP-4 inhibitors directly suppress macrophage foam cell formation, independent of increased incretins. However, this effect of DPP-4 inhibitors on T2D patients and diabetic mice has not been clarified in an *in vitro* study. In the present study, we observed remarkable suppression of foam cell formation by ex vivo treatment with a DPP-4 inhibitor in macrophages isolated from diabetic *db/db* mice and T2D patients; this effect was accompanied by a reduction of CD36 and ACAT-1 expression levels. Thus, our findings here are the first to show that a DPP-4 inhibitor has the potential to suppress foam cell formation regulated by CD36 and ACAT-1 in macrophages extracted from diabetic mice and T2D patients. Because previous studies have shown that CD36 and ACAT-1 levels are mainly associated with PI3K and PKC [17–19], a DPP-4 inhibitor can possibly regulate CD36 and ACAT-1 expressions via the PI3K/PKC signaling pathway in macrophages of diabetic mice and T2D patients. Future studies should be needed to clarify the complex networks of these unidentified pathways concerned in macrophages.

Recent studies support the crucial role of inflammation in the pathogenesis of atherosclerosis and the strong association between hyperglycemia and systemic inflammation. Because inflammation is another key factor for atherogenesis [20], there have been some *in vivo* studies of anti-inflammatory and antiatherosclerotic effects of DPP-4 inhibitors on animal models and T2D patients [8, 21]. Tremblay et al. [21] showed that plasma levels of IL-6 in T2D patients are reduced by a DPP-4 inhibitor. Sato-Asahara et al. [22] observed that serum IL-6 and monocyte IL-6 expression levels in T2D patients tended to decrease due to *in vivo* treatment with DPP-4 inhibitors. However, these researchers failed to demonstrate the suppressive effect of DPP-4 inhibitors on monocytes/macrophages in T2D patients ex vivo. We previously reported that *ex vivo* treatment with teneligliptin led to remarkable suppression of lipopolysaccharide-induced IL-6 expression levels in mouse peritoneal macrophages and human monocyte/macrophage U937 cells [11]. Therefore, we selected the proinflammatory cytokine IL-6 in this study to evaluate the effect of a DPP-4 inhibitor on the proinflammatory function of macrophages isolated from diabetic mice and T2D patients. Our findings show that in addition to suppressing foam cell formation, *ex vivo* teneligliptin treatment decreased inflammatory signals in macrophages, which may also provide support for contributing to the protection against atherosclerosis in high inflammatory conditions such as T2D.

The present study has some potential limitations in human experiments. Therapy with insulin and oral antidiabetic, lipid-lowering, and antihypertensive drugs may partly contribute to foam cell formation in monocyte-derived macrophages isolated from T2D patients. Additionally, there were some differences in terms of body weight and lipid parameters between humans and mice in this study. The lack of evaluations of these differences is another limitation in this study. Future studies are needed to clarify these differences with foam cell formation in macrophages. The mouse experiment in this study is subject to some limitations. The protein levels of regulated CD36 and ACAT-1, as well as the activity levels of ACAT-1 and other inflammatory cytokine NF-*κ*B compounds, were not evaluated. Nevertheless, the extent of foam cell formation in macrophages isolated from diabetic mice and T2D patients was enhanced in comparison with the formation in those from nondiabetic mice and healthy volunteers, respectively. Further, we observed remarkable suppression of macrophage foam cell formation by ex vivo treatment with a DPP-4 inhibitor in T2D patients, which was consistent with the results observed in the diabetic *db/db* mice. Although the number of human subjects might be insufficient in this study, our findings imply that a DPP-4 inhibitor suppresses macrophage foam cell formation on both species.

## 5. Conclusions

The present study is the first to demonstrate that a DPP-4 inhibitor has the potential to suppress foam cell formation via CD36 and ACAT-1 in macrophages isolated from diabetic *db/db* mice and T2D patients *ex vivo*. This effect is conserved across species and is worth studying further to elucidate its potential as an intervention for antiatherogenesis in T2D patients.

## Figures and Tables

**Table 1:** Characteristics and laboratory data of *db/db misty* mice and *db/db* mice at 13 weeks old.

	*db/db misty*	*db/db*	*p* value
Number	7	7	−
Food intake (g/day)	3.1 ± 0.1	4.5 ± 0.2	*p* < 0.005^★^
Final body weight (g)	25.4 ± 0.3	43.8 ± 0.6	*p* < 0.0001^★^
SBP (mmHg)	106 ± 4	109 ± 4	0.622
DBP (mmHg)	57 ± 3	61 ± 2	0.274
Total-C (mg/dL)	102 ± 3	112 ± 16	0.473
HDL-C (mg/dL)	56 ± 3	63 ± 13	0.573
Triglyceride (mg/dL)	91 ± 3	134 ± 16	*p* < 0.05^★^
Insulin (ng/mL)	0.27 ± 0.03	2.48 ± 0.32	*p* < 0.0001^★^
FBG (mg/dL)	107 ± 5	432 ± 26	*p* < 0.0001^★^
HbA1c (%)	4.2 ± 0.1	8.3 ± 0.2	*p* < 0.0001^★^
OGTT-AUC (mg/dL × hour)	276 ± 3	1215 ± 85	*p* < 0.0001^★^

SBP: systolic blood pressure; DBP: diastolic blood pressure; Total-C: total cholesterol; HDL-C: high-density lipoprotein cholesterol; FBG: fasting blood glucose; HbA1c: hemoglobin A1c; OGTT: oral glucose tolerance test; AUC: area under the curve of glucose. Results are presented as mean values ± SEM and analyzed with unpaired *t*-test. ^★^ *p* < 0.05 vs. *db/db misty* mice.

**Table 2:** Characteristics and plasma biochemical parameters of T2D patients and healthy volunteers.

	Healthy volunteers	T2D patients	*p* value
Number	4	4	—
Age (years)	51 ± 7	63 ± 9	0.329
Duration of diabetes (years)	—	19 ± 6	—
Body weight (kg)	69 ± 3	74 ± 9	0.666
BMI (kg/m^2^)	22.6 ± 0.8	26.4 ± 3.2	0.282
SBP (mmHg)	117 ± 3	113 ± 2	0.376
DBP (mmHg)	76 ± 5	70 ± 3	0.284
Total-C (mg/dL)	189 ± 6	187 ± 10	0.84
LDL-C (mg/dL)	120 ± 4	119 ± 9	0.888
HDL-C (mg/dL)	45 ± 6	39 ± 5	0.393
Triglyceride (mg/dL)	118 ± 13	149 ± 4	0.06
FBG (mg/dL)	93 ± 2	189 ± 24	*p* < 0.01^★^
HbA1c (%)	5.4 ± 0.2	8.3 ± 0.2	*p* < 0.0005^★^
Retinopathy (NDR/SDR/PPDR/PDR)	NA	2/0/0/2	—
Nephropathy (1/2/3/4/5)	NA	0/3/1/0/0	—
Macroangiopathy (CVD/CAD/PAD/CAD + PAD/none)	NA	0/0/0/2/2	—
Daily bolus insulin dose (unit)	—	36 ± 9	—
Daily basal insulin dose (unit)	—	12 ± 3	—
Total daily insulin dose (unit)	—	48 ± 12	—
Use of OADs (Met/Met + Pio/none)	0/0/4	1/1/2	0.264
Lipid-lowering drugs (statin/none)	2/2	4/0	0.103
Antihypertensive drugs (ARB/ARB + *β*-blocker/none)	0/0/4	2/1/1	0.091

T2D: type 2 diabetes; BMI: body mass index; SBP: systolic blood pressure; DBP: diastolic blood pressure; Total-C: total cholesterol; LDL-C: low-density lipoprotein cholesterol; HDL-C: high-density lipoprotein cholesterol; FBG: fasting blood glucose; HbA1c: hemoglobin A1c; NDR: no diabetic retinopathy; SDR: simple diabetic retinopathy; PPDR: preproliferative diabetic retinopathy; PDR: proliferative diabetic retinopathy; NA: not available; —: none; CVD: cerebrovascular disease; CAD: coronary artery disease; PAD: peripheral artery disease; OADs: oral antidiabetic drugs; Met: metformin; Pio: pioglitazone; ARB: angiotensin II receptor blocker. Results are presented as mean values ± SEM and analyzed with unpaired *t*-test. Categorical variables were compared by the chi-square test. ^★^ *p* < 0.05 vs. healthy volunteers.

## Data Availability

All data used to support the findings of this study are available from the corresponding author upon request.

## Supplementary Materials

Supplementary 1 Supplementary Figure 1: effects of teneligliptin on foam cell formation in monocyte-derived macrophages isolated from individual T2D patients and healthy volunteers. Human peripheral mononuclear cells were isolated from each of four healthy volunteers (a-d) or four T2D patients (e-h). The cellular lipids were extracted, and the radioactivity of the cholesterol [^3^H] oleate was determined by thin-layer chromatography. We extracted 4–5 dishes of adhered monocyte-derived macrophages isolated from each of healthy volunteers (a-d), T2D patients (e-h), and T2D patients with 10 nmol/L ex vivo teneligliptin treatment after isolation (e'-h'). The background characteristics and plasma biochemical parameters of individual T2D patients and healthy volunteers are also listed in Supplementary Table 1. Data information: results are presented as mean values ± SEM and analyzed with one-way ANOVA: ^★★★^ *p* < 0.005 vs. health volunteers. ^☆☆^ *p* < 0.01 vs. T2D without teneligliptin *ex vivo*.

Supplementary 2 Supplementary Table 1: individual characteristics and plasma biochemical parameters of T2D patients and healthy volunteers.
