# Supplementary figures and images for: A Dipeptidyl Peptidase-4 Inhibitor Suppresses Macrophage Foam Cell Formation in Diabetic db/db Mice and Type 2 Diabetes Patients
Source: Int J Endocrinol. 2018 Dec 9;2018:8458304. doi: 10.1155/2018/8458304 (PMC6304851; doi:10.1155/2018/8458304)

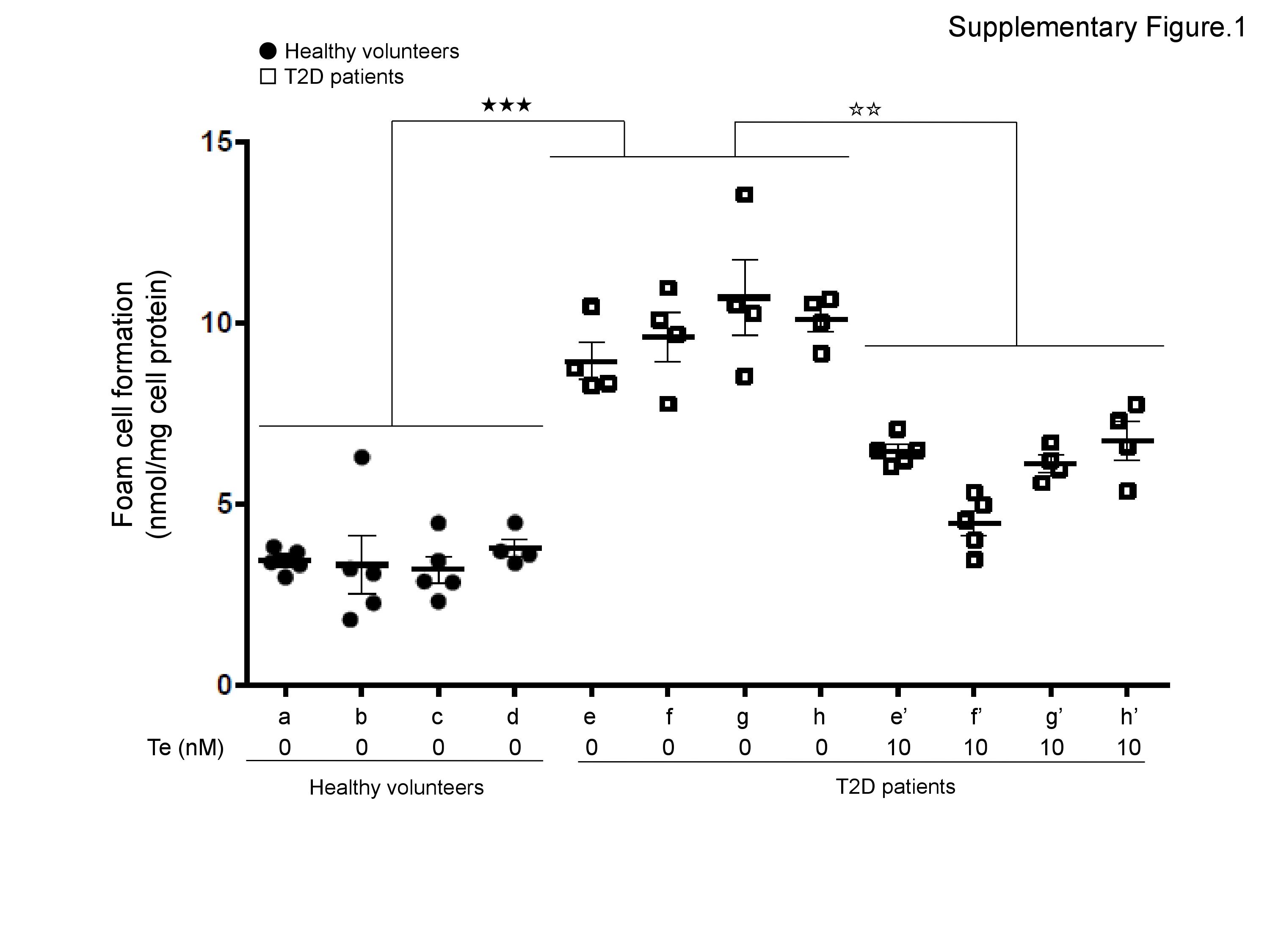

Supplement: Supplementary 1 — Supplementary Figure 1: effects of teneligliptin on foam cell formation in monocyte-derived macrophages isolated from individual T2D patients and healthy volunteers. Human peripheral mononuclear cells were isolated from each of four healthy volunteers (a-d) or four T2D patients (e-h). The cellular lipids were extracted, and the radioactivity of the cholesterol [3H] oleate was determined by thin-layer chromatography. We extracted 4–5 dishes of adhered monocyte-derived macrophages isolated from each of healthy volunteers (a-d), T2D patients (e-h), and T2D patients with 10 nmol/L ex vivo teneligliptin treatment after isolation (e'-h'). The background characteristics and plasma biochemical parameters of individual T2D patients and healthy volunteers are also listed in Supplementary Table 1. Data information: results are presented as mean values ± SEM and analyzed with one-way ANOVA: ★★★ p < 0.005 vs. health volunteers. ☆☆ p < 0.01 vs. T2D without teneligliptin ex vivo. [file 8458304.f1.jpg]

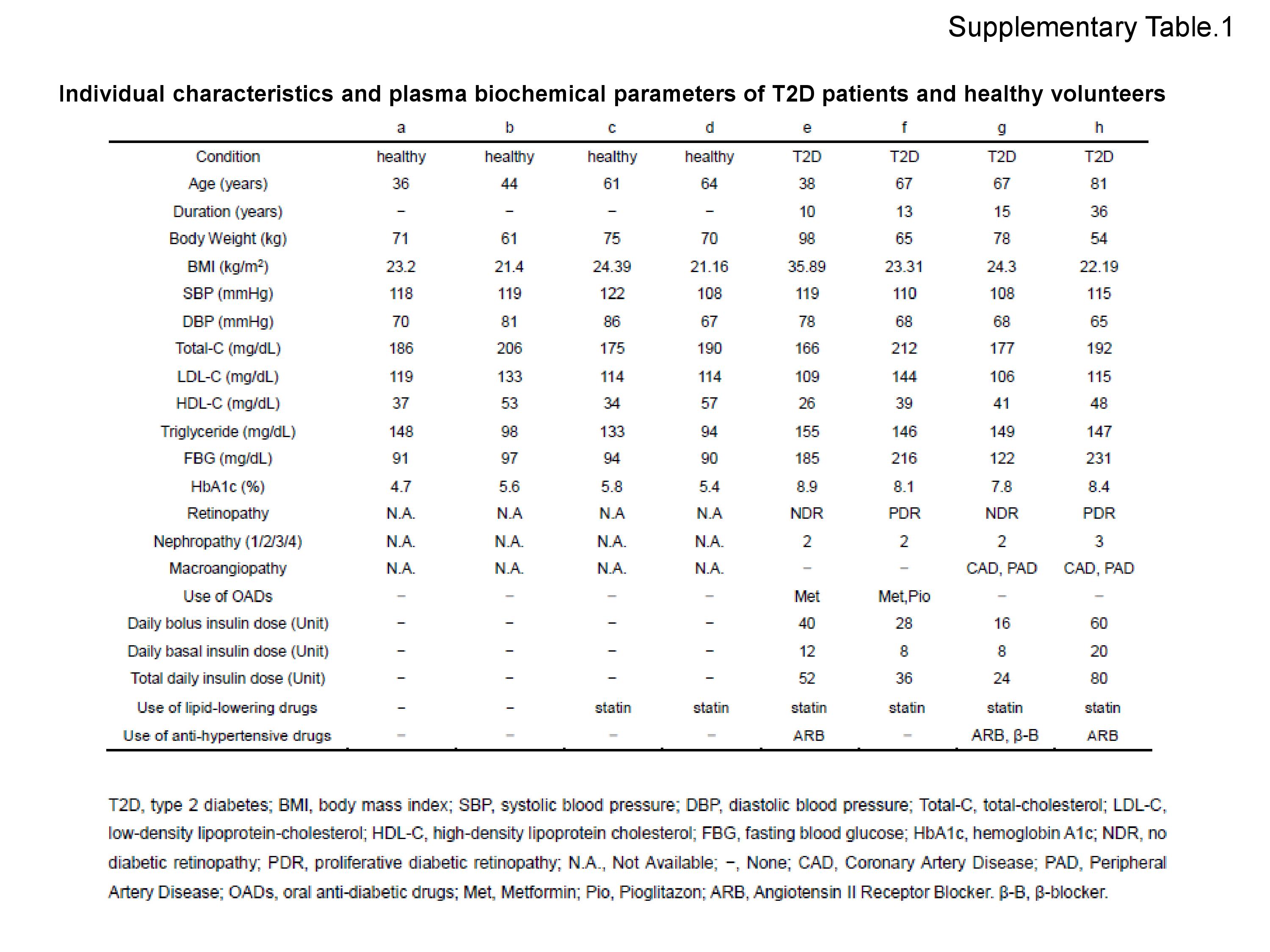

Supplement: Supplementary 2 — Supplementary Table 1: individual characteristics and plasma biochemical parameters of T2D patients and healthy volunteers. [file 8458304.f2.jpg]
